# Wearable, Knitted 3D Spacer Thermoelectric Generator with Detachable p-n Junctions for Body Heat Energy Harvesting

**DOI:** 10.3390/s24165140

**Published:** 2024-08-08

**Authors:** Samantha Newby, Wajira Mirihanage, Anura Fernando

**Affiliations:** Department of Materials, The University of Manchester, Manchester M13 9PL, UK

**Keywords:** knitted device, thermoelectric generation, textile, thermal imaging, modeling

## Abstract

Textile-based thermoelectric (TE) devices are being investigated to power smart textiles autonomously. While previous research has focused on a solid system where the required junctions are fabricated into the device, there has been limited attention given to replacing these TE systems reliably. This work looks at a newer approach to the construction and demonstration of a wearable thermoelectric structure that employs three-dimensional knitted spacers to increase the temperature difference where the TE junctions are detachable and disposable. This system features positive and negative junctions which can be removed while maintaining its excellent voltage generation in low ΔT and good Seebeck coefficients. A mathematical model simulates the potential energy outputs and maximum power points generated, which can be used to increase the device’s performance for future wearable sensing applications.

## 1. Introduction

Electronic textiles (e-textiles) are increasingly popular, and there is a drive to find ways of powering these devices autonomously. One way this can be accomplished is through thermoelectric (TE) systems, which can convert a temperature difference to electrical energy via the Seebeck effect [1]. The Seebeck effect is when positive and negative materials are connected at a junction, the p-n junction, are subjected to a temperature difference across the boundary of the device and create electrical energy through this difference [2]. To use wearable TE systems in medical, sports, and protective applications, the systems need to be flexible, lightweight, have the ability to work in low temperatures, and, if worn by humans as a garment, comfortable [3,4,5,6].

Wearable TE systems require different characteristics than those used in other applications, such as hybrid vehicles, as the temperature difference (ΔT) is much smaller [7,8]. The structure of the wearable device influences how effective the system can be, as it can give a natural temperature difference based on its thickness and makeup. A knitted three-dimensional (3D) spacer fabric is ideal because the conductive TE system can be seamlessly integrated into a structure that already has a designated thickness and density. However, while the research into advanced, material-based knitted TE systems is ongoing, researchers have not been able to account for the high electrical resistance in these systems, so the researchers demonstrate the TE device performing at higher ΔTs than what can be used for wearable technology [9,10,11,12].

3D-spacer knitted structures are being investigated for TE application because their structures have natural gaps between the boundary yarns, which allows for an added temperature difference between the skin and the atmosphere. However, there is very little research into a 3D-spacer knit structure that features knitted-in p-n wire conductive pathways. Other researchers have created the 3D structure and then embroidered, sewn, or deposited the conductive material post-fabrication [13,14,15]. Li et al. created a 3D spacer knit for TE application using PEDOT:PSS and constantan where the device was glued to the spacer-knit structure [16]. With 100 units connecting the two sides of the spacer fabric, the results showed an output voltage of around 0.5 mV at a 20 K temperature difference. Another group doped 3D spacer fabrics post-fabrication in aluminum-doped zinc oxide and silver, then threaded a conductive wire through the knit to connect the two sides, creating a device that generated a power of 0.2 µW [15]. This device did investigate a low ΔT of 10 K, with the results showing around 0.5 mV and 26 µA for voltage and current, respectively. Only one research group, Dallmann et al., used a single-fabrication method for their 3D spacer fabric, resulting in a power output of 1.78 µW [17]. However, the results for this spacer knit are captured when the ΔT is from 32 to 65 K, which is too high of a temperature difference to be worn on humans.

Yang et al. show a knitted TE device with high stretchability made from silver and silver selenide which was then sewn onto a knit structure [18]. While this device featured excellent bending capabilities, this process is not a single-knit process; it involves creating strips of TE material and manually inserting them into the knitted fabric. This research does feature a good voltage output of four mV at a ΔT of 15 K. Kim et al. also embedded conductive yarn into a knit structure [19]. They used wet-spun graphene to create the p-n junctions, with a low voltage output of 0.2 mV at a 10 °C temperature difference and no reported power output.

Also, while a knitted system is seamless, there is a problem when the junctions wear down or disintegrate. If this happens, the fabric will need to be disposed of and a new one made, creating waste products. Some researchers have encapsulated their advanced materials to protect them from wearing down [20]. Dong et al., for example, created their TE device and then encapsulated it with a silica gel, polydimethylsiloxane, and polymethylmethacrylate [21]. This kept the device from being damaged during washing and maintained its high flexibility. However, this device was fully encapsulated, requiring copper connections added post-fabrication and creating areas where the device could be damaged. Therefore, creating a practical, knitted, wearable device featuring detachable and disposable parts is important to the feasibility of wearable TE systems.

In this work, we present a wearable 3D-spacer garment and TE system featuring detachable and disposable p-n junctions. This 11-junction device offers good output voltage, current, and Seebeck coefficients at low-temperature applications. This device’s facile manufacturing process and its detachable parts increase its reliability as a practical system for long-term wearable applications.

## 2. Materials and Methods

### 2.1. Materials

The knitted 3D-spacer structure was fabricated with polypropylene yarn (purchased from Uppingham Yarns, Uppingham, UK) and acrylic yarn (purchased from SageZander Ltd., Congleton, UK). The p-n junctions were produced by using K-type thermocouples with Chromel and Alumel as the p-type and n-type material, respectively. 6 mm nickel-coated snaps from Hemline were used to facilitate the detachable p-n junctions, and 100% cotton fabric (purchased from Abakhan, Manchester, UK) was used to support the detachable p-n junctions.

### 2.2. Fabrication of TE Device

**Figure 1:**
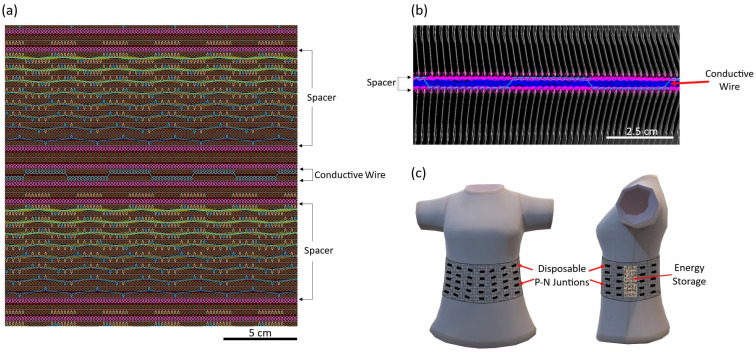
Device’s (**a**) knit notation, (**b**) knitting course showing the wire integration, and (**c**) schematic when applied to a shirt.

The TE device was fabricated from a knitted 3D-spacer fabric, with p-n junctions placed on alternate surfaces of the textile. The 3D spacer was knitted on a CAD-operated Shima Seiki N.SVR123 electronic flat-knitting machine, with a conductive wire knitted in one course with exterior points along both sides of the device. The knit notation can be seen in Figure 1a,b, which shows the wire integration within the knit structure in light blue. The exterior points were cut and soldered to the complementary snap and then sewn in place for stability. The thermocouple junctions were used as the p-n junctions, soldered to the nickel snaps, and sewn onto cotton fabric. The resulting device was 230 mm × 86 mm × 5 mm, with 11 p-n junctions evenly spaced along the conductive wire’s course, and could be fabricated into a garment, as depicted in Figure 1c.

### 2.3. Mathematical Modeling

**Figure 2:**
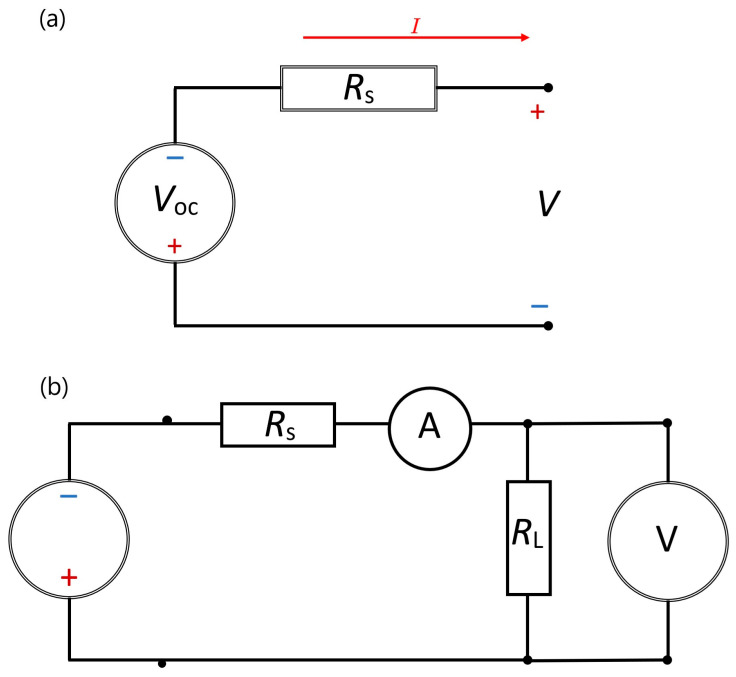
TEG representation of (**a**) the equivalent electrical representation and (**b**) the test circuit.

In a TE device, the basic representation, the movement of the device’s temperature difference, and the test circuit are shown in Figure 2. The current (*I*) and voltage (*V*) characteristics are represented through Kirchhoff’s voltage law being applied to this circuit [22].

Kirchhoff’s equation can be applied to the above circuit, consisting of one thermoelectric element and considering *Rs* as the internal resistance of thermoelectric circuit and *R_L_* as the load resistance:

(1) $$V-V_{OC}+IR_{S}=0,$$

Equation (1) can also be written as

(2) $$I=-\frac{V}{R_{S}}+\frac{V_{OC}}{R_{S}}$$

where $R_{S}$ is the TEG source resistance (Ω) and $V_{OC}$ is the TEG open-circuit voltage (volts). For a TE device, the relationship between the voltage and the temperature difference ΔT across the TEG can be given as

(3) $$V_{OC}=\alpha \Delta T$$

where α is the Seebeck effect.

**Figure 3:**
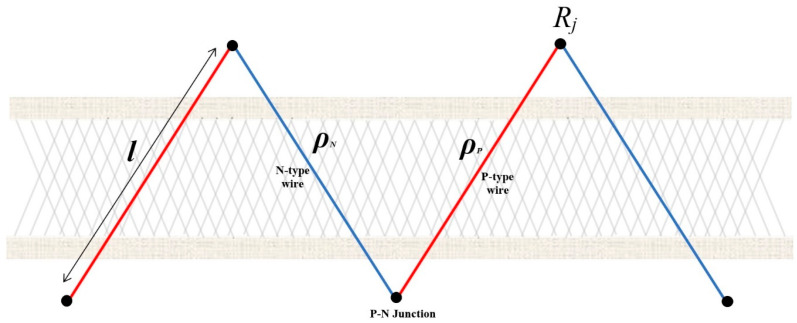
Thermocouple network for TE device.

Using the thermocouple network depicted in Figure 3, the equation for the circuit path can be written as

(4) $$R_{S}=n\left[l\left(\frac{\rho_{P}}{A_{P}}+\frac{\rho_{N}}{A_{N}}\right)+2R_{j}\right]$$

where the distance between thermocouple junctions is *l*, the resistivity of the n-type thermocouple wire is $\rho_{N}$, the resistivity of the p-type thermocouple wire is $\rho_{P}$, the cross-sectional area of the n-type wire is $A_{N}$, the cross-sectional area of the p-type wire is $A_{P}$, the number of p-n junctions is *n*, and the circuit path junction resistance is $R_{j}$. Then, combining Equations (2)–(4), the thermoelectric model of the TEG can be expressed as

(5) $$I=-\frac{V}{n\left[l\left(\frac{\rho_{P}}{A_{P}}+\frac{\rho_{N}}{A_{N}}\right)+2R_{j}\right]}+\frac{\alpha \Delta T}{n\left[l\left(\frac{\rho_{P}}{A_{P}}+\frac{\rho_{N}}{A_{N}}\right)+2R_{j}\right]}$$

Considering the flow of current *I* through the load resistor *R_L_*, the following equations can also be written,

(6) $$I=\frac{\alpha \Delta T}{n\left[l\left(\frac{\rho_{P}}{A_{P}}+\frac{\rho_{N}}{A_{N}}\right)+2R_{j}\right]+R_{L}}$$

(7) $$V=\left(\frac{\alpha \Delta T}{n\left[l\left(\frac{\rho_{P}}{A_{P}}+\frac{\rho_{N}}{A_{N}}\right)+2R_{j}\right]+R_{L}}\right)R_{L}$$

where the Seebeck effect depends on the number of high–low temperature p-n junction couples.

## 3. Experimental

To characterize the TE performance of this device, a Benchmark 230 V hotplate was used to simulate the radiated body temperature, or skin temperature, with the atmospheric temperature in the room acting as the unheated side. Two wires were soldered to the outermost snaps in the system and attached to the 10-bit (±1 V) Voltage Input Phidget (purchased from Active Robots, Bath, UK. To maintain a heated temperature representing the human body’s waste heat, a K-type thermocouple was fixed to the hotplate below the device to monitor the heated temperature and maintain a fixed temperature during testing. A second thermocouple was placed on the exposed side of the knitted device to monitor the cooler side of the device. The atmospheric temperature and humidity in the room during the tests were 24.4 °C and 53.8%, respectively. A TIM thermal imaging camera (purchased from Micro-Epsilon, Birkenhead, UK) was used to analyze the surface temperature of the knitted spacer device when heated from 30.1 to 44.8 °C and cooled back to 30.1 °C.

To test the device on an individual, the device was placed at three locations on the body, the thigh, stomach, and upper arm, and then taped into place with a k-type thermocouple between the device and the skin to determine the heated temperature. The device was attached to the 10-bit (±1 V) Voltage Input Phidget. The atmospheric temperature and humidity in the room during the tests were 24.9 °C and 51.2%, respectively.

## 4. Results and Discussion

### 4.1. Device Structure

**Figure 4:**
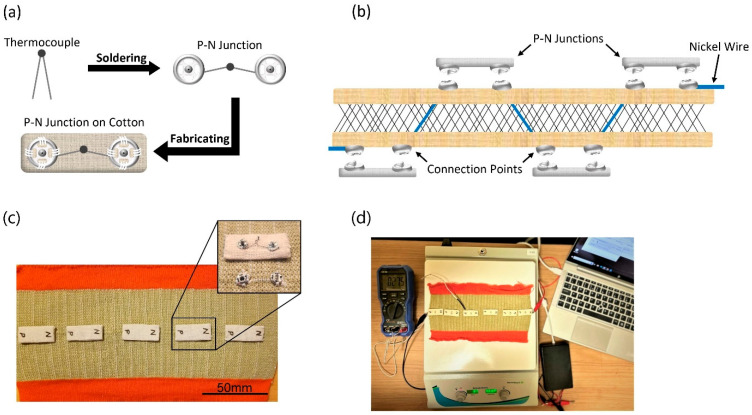
Knitted 3D-spacer fabric with (**a**) a schematic of the detachable p-n junction, (**b**) a schematic of the knitted spacer structure with conductive wires and snaps, (**c**) an image of the knitted structure with detachable p-n junction, and (**d**) the experimental setup on a hot plate.

The knitted spacer structure resists heat transfer and can maintain a higher ΔT than a fabric that is only one layer thick. Conductive k-type thermocouple wires, made from Alumel and Chromel, were knitted through the structure in a single course, with exterior points found on both sides of the fabric [23]. These conductive wires were chosen for their knittability and the strength of the welded p-n junction (Figure S1). The thermocouple legs knitted into the device were 20 mm in length and were cut at 10 mm increments to be soldered to the snap closures, which are common sewing notions. Nickel-plated snap closures are electroconductive and ideal separators if the TE device needs to be detached or replaced, and they maintain low electrical resistance (Figure 4a). By interrupting the electrical circuit of the TE device with the snap, the structure can maintain its electrical resistance while allowing the p-n junctions to be detached and replaced when necessary, as shown in schematic Figure 4b. The final TE device used in this research had 11 p-n junctions evenly spaced along both sides of the conductive wire’s knitted course, with six pairs of junctions reaching from the cold to the hot side of the device, Figure 4c. The initial test of the device on a heated plate is shown in Figure 4d.

### 4.2. Thermoelectric Capabilities

The electrical resistance of the TE device is critical to the results, as it impacts the voltage output, the Seebeck coefficient, and the generated current. The overall resistance of the device measured 30.87 ohms (Ω) before washing and 33 Ω after 10 wash cycles. Due to the device’s detachable junctions, the overall resistance cannot vary when the junctions are replaced to maintain the device’s TE efficiency. Therefore, the junctions were replaced 100 times, and the resistance was taken each time (Figure S2, Table S2). These results showed that the resistance remained under 80 Ω but did vary due to altered surface contact between the different snaps. However, the snap notions did not damage the nickel coating, indicating long-life application. The device’s resistance was also captured during a bending test to understand how deformation would affect the resistance of the device. It showed that the device’s resistance reduced during bending and then increased when the device was unbent (Figure S3). This is because of the contact resistance increase during the bend as the garment contracted around the snaps.

**Figure 5:**
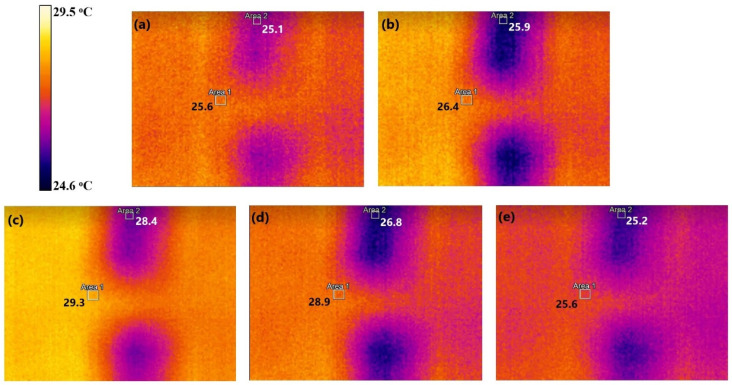
Thermal imaging of the device during a heat transfer test at (**a**) starting temperature, (**b**) midway through heating, (**c**) the hottest temperature, (**d**) midway through cooling, and (**e**) the final temperature.

When a TE junction is exposed to a temperature difference, there is a thermal diffusion of electrons from the hotter side to the cooler side because the increased entropy in the heated electrons transports them to the colder side [6]. The electrons also move from the n-type material, which has a surplus of electrons, to the p-type material, which is deficient in electrons, creating a flow of energy. However, if the heat transfer occurs through the device, it cannot maintain the temperature difference and gradually decreases electron transport. Thus, direct thermal diffusion of the device can severely limit the device’s TE capabilities. A knitted 3D-spacer structure can stop this effect by providing thermal insulation. Figure 5 shows the thermal imaging of the device’s surface when heated to a steady-state temperature and the overall heat transfer from the hot side to the cooler side. Area 1 is the knit fabric and Area 2 is the replaceable p-n junctions. During the heating period, when the temperatures were at a steady state, the results show that the spacer fabric managed to maintain a temperature difference of 15.5 °C between its hot side and cooler side (Supplementary Video S1). These results show the spacer knit’s excellent thermal insulation, allowing its TE performance to remain constant through different human skin temperatures and activities.

**Figure 6:**
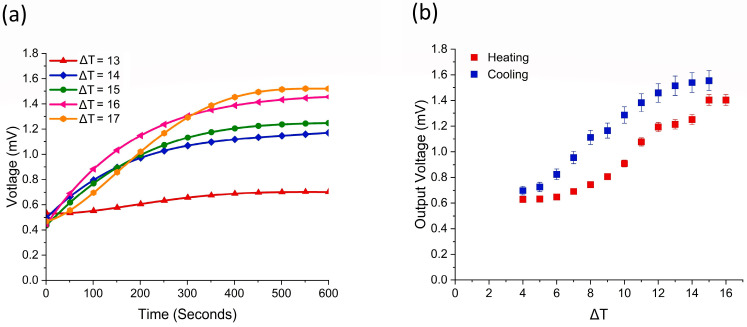
Thermoelectric characteristics: (**a**) voltage over time at varying ΔT and (**b**) device heated and cooled.

The temperature difference in the TE system creates an output open-circuit voltage that can be analyzed. To test the capabilities of this device, it was connected to a multimeter and heated to temperatures from 36 °C to 40 °C, resulting in ΔTs of 13 to 17 degrees, respectively. The device was heated from room temperature, 23 °C, to different temperatures and held until a steady output voltage was reached, as seen in Figure 6a. The resulting voltages ranged from 0.7 to 1.52 mV for ΔT 13 to 17 °C, respectively. The test shows that the highest open-circuit output voltage occurs with the highest ΔT, proving that the thermoelectric capabilities are linked to the temperature difference. 

Subsequently, the device was tested to analyze the device’s heating and cooling properties. The device was heated from room temperature to just below 40 °C with a ΔT from 4 to 17 degrees, as shown in Figure 6b. The overall output open-circuit voltage increased with the ΔT but reached its highest voltage, 1.56 mV, at a ΔT of 14 after the heating was turned off. This shows a slight delay between heating the device and the improved voltage from the system. When the device cooled, it maintained a higher voltage than when it was heated, most likely because of thermal inertia in the device.

**Figure 7:**
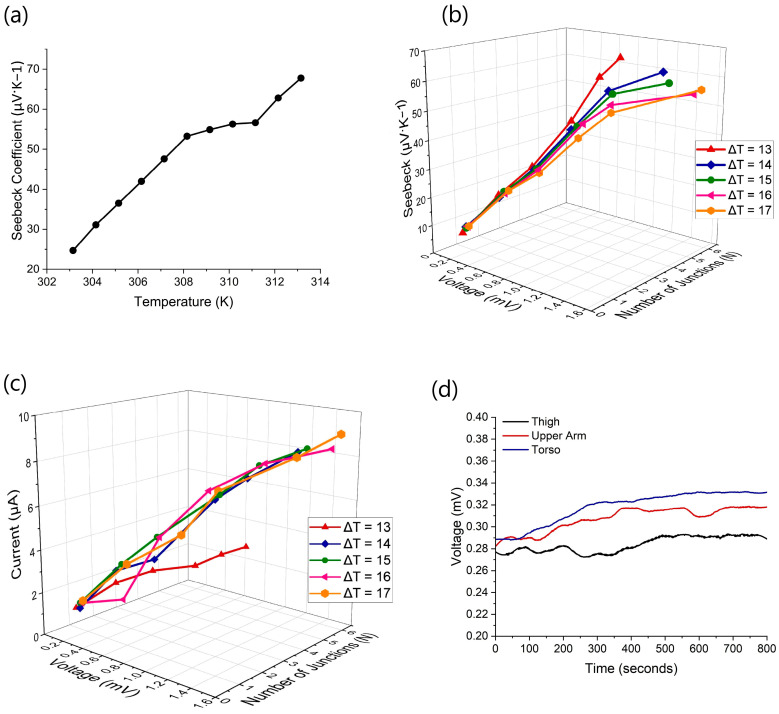
Thermoelectric performance of (**a**) the Seebeck coefficient with regards to temperature, (**b**) the change in voltage, Seebeck coefficient, and number of p-n junctions at varying ΔT, (**c**) the change in voltage, current, and number of p-n junctions at varying ΔT, and (**d**) voltage from the human body.

The Seebeck coefficient (α) captures the efficiency of the TE materials and system, determined from the relationship, α = ∆V/∆T [2]. The p-type material has a Seebeck coefficient of 11.54 µV·K−1 and the n-type material, −6.82 µV·K−1 at 295.15 K. The device’s Seebeck coefficient was determined for temperatures 303.15 to 313.15 K in single-degree increments, with results measured across a range varying from 24.7 to 67.7 µV·K−1, respectively, as shown in Figure 7a. This proves that the Seebeck coefficient is temperature-dependent and increases with the overall ΔT applied to the device. For wearable applications, temperatures up until 312 K are suggested, giving a Seebeck coefficient of 62.79 µV·K−1, higher than other examples of knitted 3D-spacer TE devices and at lower temperatures [15,16,24].

To analyze the efficiency of the TE system with varying numbers of paired junctions, the device was tested at different temperature differences with one to six paired junctions being heated. This resulted in different output voltages and different Seebeck coefficients. The device was again heated to 36, 37, 38, 39, and 40 °C, resulting in a ΔT from 13 to 17 degrees, respectively, as seen in Figure 7b. These results show the higher number of paired junctions created a higher output voltage and Seebeck coefficient. For one pair of junctions, the resulting output voltage and Seebeck coefficient ranged from 0.08 mV and 5.13 µV·K−1 at ΔT 13 as compared to 0.16 mV at 8.3 µV·K−1 at ΔT 17. When all junctions were used, the output voltage increased from 0.71 mV to 1.52 mV at ΔT 13 and 17, respectively. However, the Seebeck coefficient decreased from 62.7 µV·K−1 to 55.7 µV·K−1 at ΔT 13 and 17, respectively. This is due to the ΔT value increasing faster than the ΔV value and having a higher impact on the resulting coefficient. This confirms that a lower ΔT allows the device to perform more efficiently than a higher ΔT.

Similar tests were performed to analyze the performance of the device, with different numbers of paired junctions. Figure 7c shows that the current and voltage increased with the number of junctions and the ΔT. The higher the number of paired junctions and ΔT, the higher the current, with the highest current being 9.04 µA for all junctions maintained at 17 °C temperature difference. This generated current is higher than other reported wearable TE systems that use a similar number of junctions, as seen in Table 1, due to the metal in the conductive paths and the tight connection between the snaps in the detachable system.

After confirming the capabilities of the device on a heater, the device was tested on the human body. The three areas tested, thigh, upper arm, and torso, did not reach the anticipated 36–40 °C described in the other literature [5,27]. The skin’s temperature reached 31.1 °C, 32.5 °C, and 32.8 °C for each respective area. This may be due to the locations chosen to test the device and because the radiated body temperature of a human is different from the core temperature. This is because the skin’s epidermis acts as a barrier, reducing the skin’s temperature more than the expected radiated temperature. Therefore, the overall performance of the device was lower than expected because of the lower ΔT. The results were recorded from the initial placement of the device until a steady-state output voltage was achieved, as seen in Figure 7d. The thigh demonstrated a ΔT of 6.2 °C and resulted in a voltage output of 0.29 mV, the upper arm had a ΔT of 7.6 degrees and an output voltage of 0.32 mV, and the torso had a ΔT of 7.9 degrees and an output voltage of 0.33 mV. The torso offered the greatest output voltage and should be considered a prime location for TE wearable devices. For each area, the Seebeck coefficient was calculated at 48.89 µV·K−1, 42.43 µV·K−1, and 41.88 µV·K−1 for the thigh, upper arm, and torso, respectively.

### 4.3. Analysis with Simulations

**Figure 8:**
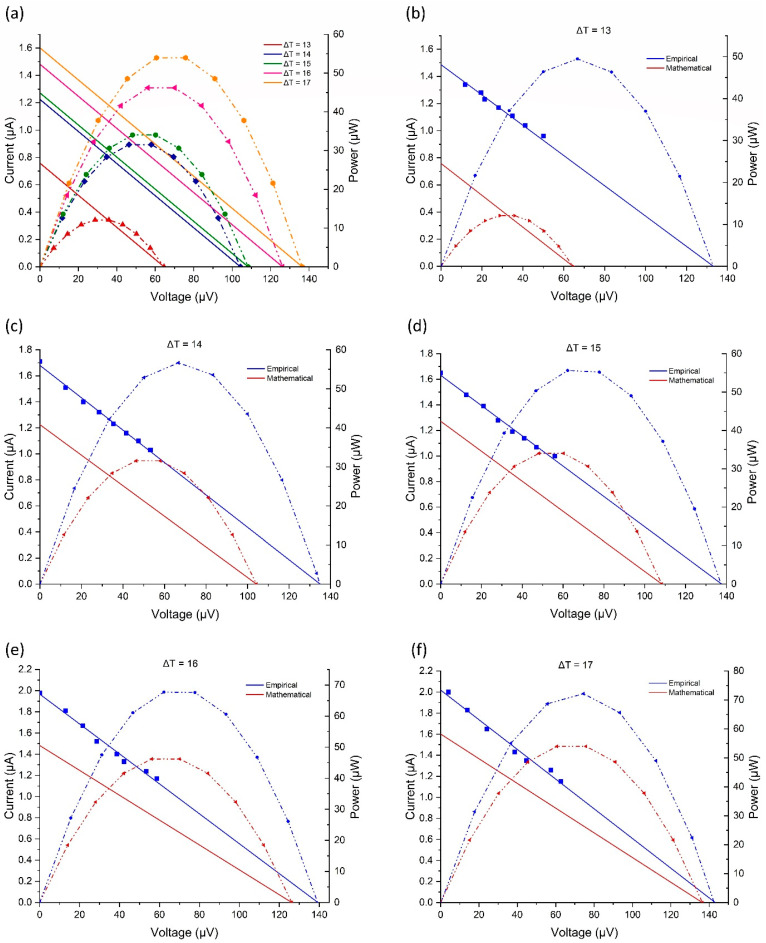
(**a**) Mathematical modeling of voltage vs current lines and power curves. Comparison of the voltage vs current lines and power curves of the empirical and mathematical data for (**b**) ΔT of 13, (**c**) ΔT of 14, (**d**) ΔT of 15, (**e**) ΔT of 16, and (**f**) ΔT of 17.

Using Equations (6) and (7) from the modeling section, a linear relationship between the simulated current and the voltage can be graphed, as shown in Figure 8a. From this, a power curve can be determined, and a maximum power point (P_max_) can be found for the single paired junction at different temperature differences. With an increase in ΔT, the current and voltage increase, raising the P_max_. The calculated P_max_ for ΔTs from 13 to 17 vary from 12.08 to 53.96 µW, respectively; see Figure 8a.

To validate the mathematical model, the device’s current and voltage were empirically measured at varying ΔT with different $R_{L}$. Figure 8b–f compares the empirical data from a single paired junction heated to different ΔTs with the mathematical model’s generated information. The empirical data is semi-linear, not linear like the mathematical model, due to heat trapped in the device, heat leakage, humidity, and other environmental factors. At each ΔT, the empirical data performed better than the model. With ΔT of 13 degrees, Figure 8b, the device had an empirical p_max_ 37 µW higher than predicted. Similarly, ΔT 14, 15, 16, and 17 empirically had higher p_max_ results of 24.5, 22, 21, and 18 µW, respectively. As the ΔT increased, the percentage error between the empirical and mathematical data decreased, and the two voltages drew together. This shows that the mathematical model became more accurate at higher temperatures. This may be because the detachable contact points in the system swell when heated, reducing the contact resistance in the device.

The relationship, while different, between the two datasets shows that the mathematical model does represent a version of the device’s performance and can be used to calculate potential voltage, current, and power generation. This comparison demonstrates that the device performs better than mathematically predicted but is relatively accurate at ΔT 17.

## 5. Conclusions

A flexible, knitted 3D-spacer fabric was successfully designed and demonstrated, with detachable and disposable parts that can be seamlessly knitted as part of a wearable sensing device. Thermal imaging confirmed that some heat is transferred through the knitted spacer structure, which needs to be controlled. However, it maintains the temperature difference across the TE configuration and results in sufficient output voltage. This demonstrates that knitted 3D-spacer structures are ideal for TE wearable devices, as they offer higher ΔT than single-layer structures. While this device features detachable junctions, the contact resistance between the snaps that hold the junctions onto the knitted device is low enough that there is no change in the resulting voltage or Seebeck coefficients generated by low ΔT. This 11-junction system produces a 1.52 mV open-circuit voltage of electrical energy, a current of 9.05 µA, a maximum power point of 70 µW, and a Seebeck coefficient of 62.79 µV·K−1 at 312 K. The device offers facile fabrication methods, and its disposable parts do not detract from its performance, making it a viable alternative to the current systems that require the entire system to be replaced if something breaks down.

## Figures and Tables

**Table 1:** Comparison of the wearable TE Systems.

Device	Material	Junctions	ΔT (K)	Voltage (mV)	Current (µA)	Max Power Point (µW)
Coated Yarn [9]	PEDOT:PSS/MWCNTs/Bi_2_Te_3_	9	20	2.19	4.7	2.64 × 10^−3^
Conductive strips sewn into Knit Fabric [18]	Ag/Ag_2_Se strips	6	40	13	215	0.9
Fiber Sewn into Knit Fabric [19]	HrGO/MrGO	1	20	0.2	-	-
Wristband with sewn conductive yarn [20]	CNT yarn with Ag NW layer	~240	19	20	600	250
Knitted Yarn Dip-Coated in conductive material [25]	CNT/PVP	6	20	0.5	-	-
Flexible Mesh Fabric [26]	Bi_2_Sb_0.3_Te_2.7_/Bi_0.5_Sb_1.5_Te_3_	12	3.7		1.8	1.4 × 10^−3^
Knitted Structure with Disposable Junctions [This work]	Alumel and Chromel	11	17	1.52	9.05	70

## Data Availability

The dataset is available upon request from the authors.

## Supplementary Materials

The following supporting information can be downloaded at: https://www.mdpi.com/article/10.3390/s24165140/s1, Figure S1: (**a**) SEM images of p-n junction at 40x magnification. Elemental analysis with (**b**) Alumel and (**c**) Chromel; Table S1: Elemental Composition of Alumel and Chromel; Figure S2: Resistances of System after Replacing p-n Junctions 100 Times; Table S2: Resistance of System after Replacing p-n Junctions; Figure S3: Bending cycle of the device and its resulting resistance; Table S3: Resistance and Conductivity of System Parts; Video S1: Thermal Imaging Video of Experiment with Heating and Cooling of Device.
