# Supplementary material for: Wearable, Knitted 3D Spacer Thermoelectric Generator with Detachable p-n Junctions for Body Heat Energy Harvesting
Source: Sensors (Basel). 2024 Aug 8;24(16):5140. doi: 10.3390/s24165140 (PMC11359260; doi:10.3390/s24165140)
Supplement: Supplementary file 1 [file sensors-24-05140-s001.zip › sensors-3115800-supplementary.pdf]

Supporting Information

# Analysis of a Wearable, Knitted 3D Spacer Thermoelectric Generator with Detachable p-n Junctions for Body Heat Energy Harvesting

Samantha Newby, Wajira Mirihanage, Anura Fernando *

The structure morphology and elemental composition of the p-n junction were analyzed with a Quanta FEG-250 SEM + EDX system. The junction shows a strong connection between both of the wires which results in a secure electrical resistance and low resistance, as seen in **Figure S1a**.

In order to confirm the materials used in the thermocouple, elemental analysis was carried out by an EDX system attached to the SEM. When the elemental analysis of the different junctions was performed, the breakdown showed that both the n-type and p-type material were exactly what they should have been, as shown in **Figure S1b/c** and **Table S1**. The traces of chlorine and manganese on the Chromel may have been deposited on there through handling and touching other material.

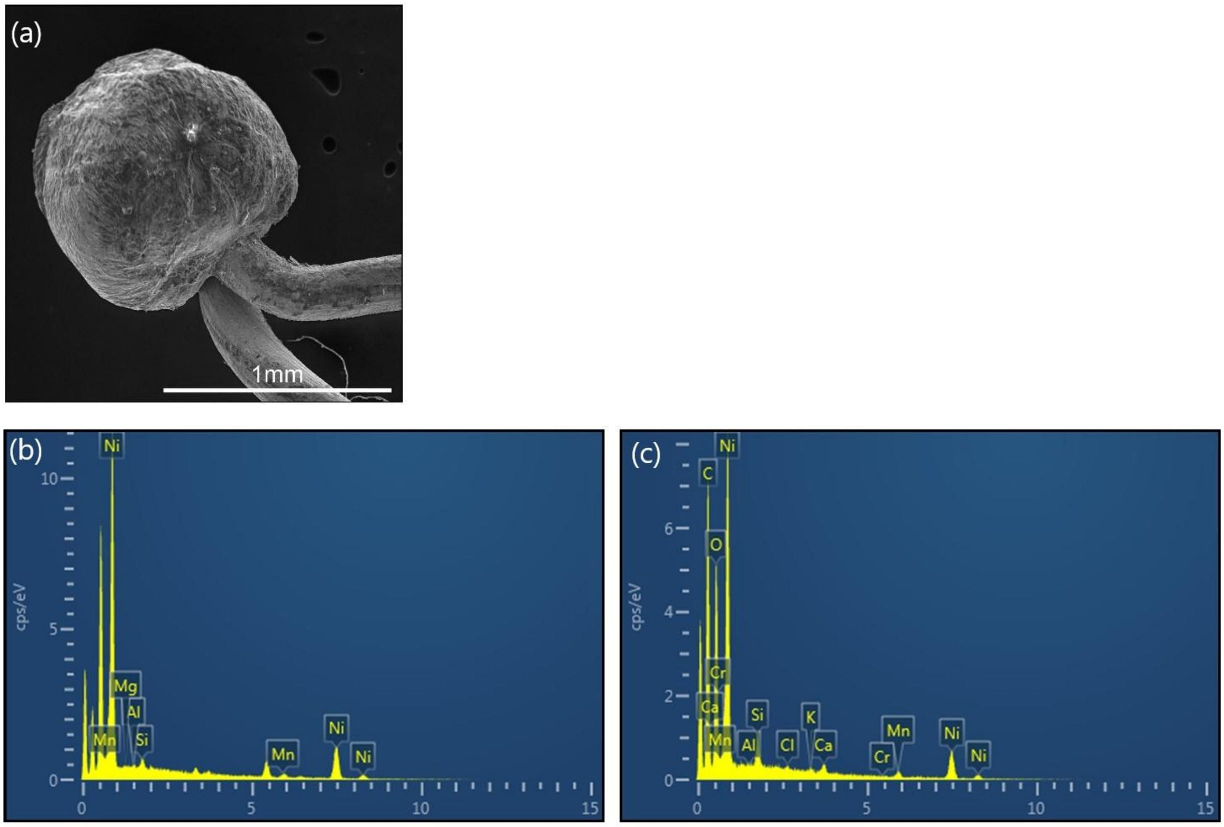


**Figure S1**. (a) SEM images of p-n junction at 40x magnification. Elemental analysis with (b) Alumel and (c) Chromel.

**Table S1**. Elemental Composition of Alumel and Chromel.

| **Material** | **Elements** | **Statistical Weight %** |
|---|---|---|
| Alumel | Nickel | 91.15 |
| | Manganese | 5.25 |
| | Silicon | 2.77 |
| | Aluminum | 0.51 |
| | Magnesium | 0.32 |
| Chromel | Nickel | 71.22 |
| | Chromium | 3.26 |
| | Oxygen | 20.75 |
| | Aluminum | 0.11 |

| | | |
|---|---|---|
| | Silicon | 2.9 |
| | Sulfur | 0.4 |
| | Chlorine | 0.61 |
| | Manganese | 0.75 |

When the electrical resistance of the device was tested, it was also tested for replacing the snaps, simulating what may happen during washing or replacing broken junctions, to see if it would affect the overall electrical resistance. The snaps were replaced 100 times, and the resistance captured each time, as shown in Figure S2 and Table S2. The overall resistance did not go over 80 Ω.

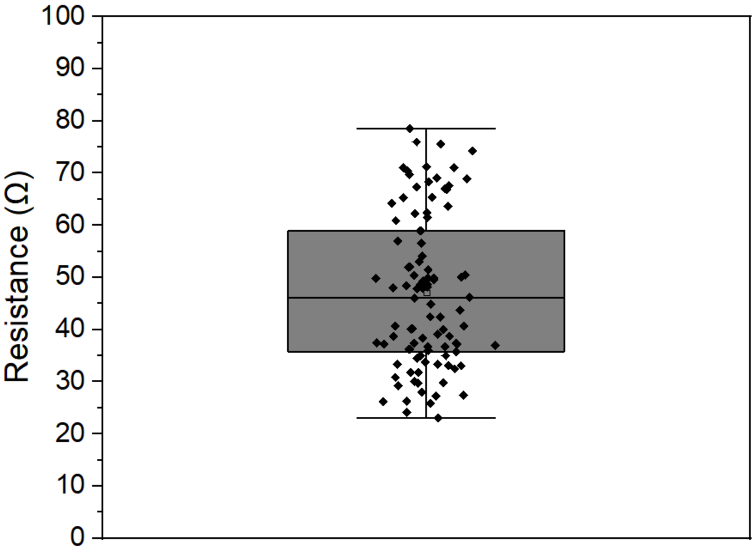


**Figure S2.** Resistances of System after Replacing p-n Junctions 100 Times.

**Table S2.** Individual Resistance of System after Each Replacement of p-n Junctions.

| Resistance (Ω) | | | |
|---|---|---|---|
| 30.87 | 33.39 | 46.10 | 35.71 |
| 37.44 | 76.01 | 36.98 | 67.61 |
| 65.35 | 78.62 | 47.85 | 36.22 |
| 50.08 | 37.24 | 49.60 | 27.23 |
| 26.21 | 34.49 | 50.48 | 50.40 |
| 65.35 | 31.81 | 37.49 | 62.39 |
| 52.97 | 49.86 | 36.73 | 49.32 |
| 47.93 | 33.09 | 35.04 | 69.00 |
| 51.47 | 29.30 | 33.35 | 58.97 |
| 67.00 | 58.97 | 74.21 | 33.09 |
| 40.73 | 38.71 | 37.17 | 28.05 |
| 49.85 | 36.24 | 63.60 | 62.27 |
| 42.48 | 33.77 | 49.92 | 29.83 |
| 35.00 | 71.10 | 26.24 | 35.96 |
| 48.43 | 49.85 | 37.44 | 52.09 |
| 48.14 | 23.13 | 38.75 | 54.11 |
| 44.85 | 48.60 | 40.07 | 27.48 |
| 42.44 | 46.13 | 71.11 | 60.86 |
| 40.02 | 43.66 | 31.81 | 47.90 |
| 30.08 | 68.31 | 32.51 | 64.24 |
| 67.32 | 69.01 | 71.24 | 61.51 |

| | | | |
|---|---|---|---|
| 25.87 | 69.71 | 52.00 | 36.70 |
| 66.91 | 70.41 | 38.42 | 56.94 |
| 40.62 | 48.73 | 39.14 | 56.60 |
| 29.67 | 75.60 | 40.20 | 24.20 |

The bending resistance of the device was taken and showed that the device performed better when bent, than when flat, as seen in Figure S3.

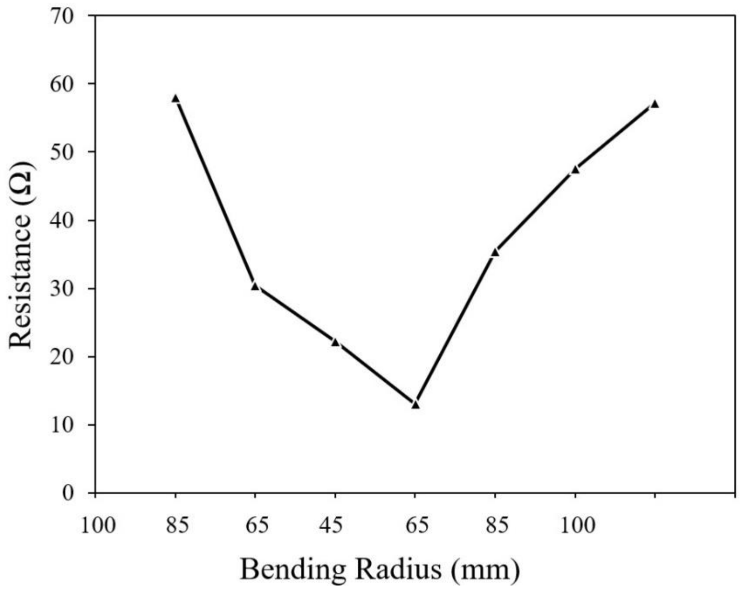


**Figure S3**. Bending cycle of the device and its resulting resistance.

In order to do an accurate analysis on the device and create mathematically simulated results, accurate electrical resistance of each part of the device was taken. This is shown in Table S4.

**Table S3.** Resistance and Conductivity of System Parts.

| **Part of System** | **Resistance (Ω)** |
|---|---|
| N-Type Nickel Wire | 0.32 |
| P-Type Nickel Wire | 0.35 |
| Thermocouple | 0.26 |
| Snap Side 1 | 0.198 |
| Snap Side 2 | 0.186 |
| Paired Snap | 0.517 |
| Single p-n Junction with Soldering | 0.650 |
| Full System | 30.872 |

When conducting thermal image analysis of the device when heated and cooled, it was captured on video the overall heat transfer rate from the hot plate through the knitted 3D spacer. This can be seen in the Video S1. Where the snapped junctions were placed shows reduced thermal temperatures, showing that less heat was penetrating through in those areas. This is due to the excess fabric needed to hold the snapped thermocouple junctions.

Video S1.mp4 (https://youtu.be/CSNvq63uM0w)
**Video S1.** Thermal Imaging Video of Experiment with Heating and Cooling of Device.
